# Novel rotatable tabletop for total-body irradiation using a linac-based VMAT technique

**DOI:** 10.1186/s13014-019-1445-3

**Published:** 2019-12-30

**Authors:** Christoph Losert, Roel Shpani, Robert Kießling, Philipp Freislederer, Minglun Li, Franziska Walter, Maximilian Niyazi, Michael Reiner, Claus Belka, Stefanie Corradini

**Affiliations:** Department of Radiation Oncology, University Hospital, LMU Munich, Marchioninistr. 15, 81377 Munich, Germany

**Keywords:** Total body irradiation, Total marrow irradiation, Volumetric modulated arc therapy (VMAT), Intensity modulated radiotherapy (IMRT), Leukemia, Tabletop, Positioning device, Treatment time, Tomotherapy

## Abstract

**Background:**

Volumetric Modulated Arc Therapy (VMAT) techniques have recently been implemented in clinical practice for total-body irradiation (TBI). To date, most techniques still use special couches, translational tables, or other self-made immobilization devices for dose delivery. Aim of the present study was to report the first results of a newly developed rotatable tabletop designed for VMAT-TBI.

**Methods:**

The VMAT-TBI technique theoretically allows the use of any standard positioning device at the linear accelerator. Nevertheless, the main problem is that patients taller than 120 cm cannot be treated in one position due to the limited cranial-caudal couch shift capacities of the linac. Therefore, patients are usually turned from a head-first supine position (HFS) to a feet-first supine position (FFS) to overcome this limitation. The newly developed rotatable tabletop consists completely of carbon fiber, including the ball bearing within the base plate of the rotation unit. The patient can be turned 180° from a HFS to a FFS position within a few seconds, without the need of repositioning.

**Results:**

The first 20 patients had a median age of 47 years, and received TBI before bone marrow transplantation for acute myeloid leukemia. Most patients (13/20) received a TBI dose of 4 Gy in 2 fractions, twice daily. The mean number of applied monitor units (MU) was 6476 MU using a multi-arcs and multi-isocenter VMAT-TBI technique.

The tabletop has been successfully used in daily clinical practice and helped to keep the treatment times at an acceptable level. During the first treatment fraction, the mean overall treatment time (OTT) was 57 min. Since no additional image guidance was used in fraction 2 of the same day, the OTT was reduced to mean 38 min.

**Conclusions:**

The easy and reproducible rotation of the patient on the treatment couch using the rotatable tabletop, is time-efficient and overcomes the need of repositioning the patient after turning from a HFS to a FFS position during VMAT TBI. Furthermore, it prevents couch-gantry collisions, incorrect isocenter shifts and beam mix-up due to predicted absolute table coordinates, which are recorded to the R + V system with the corresponding beams.

## Background

For the delivery of total-body irradiation (TBI), different approaches regarding the optimal TBI technique have been adopted in the past. Among the most frequent radiation techniques, are the use of a large source-to-surface distance (SSD) to treat the patient with one open field, or the use of a translational couch technique, where the patient is transported slowly through the radiation beam [1, 2]. A survey of the European Group of Blood and Marrow Transplantation (EBMT) confirmed this extreme heterogeneity of adopted TBI techniques [3]. The study found not only significant variations in the total dose, fractionation, dose rate, and beam energy, but also completely different irradiation techniques and types of immobilisation. In the 57 European centres who responded the survey, 11 significantly different TBI modalities were described [3]. Usually, the choice of equipment relays on the individual experience of the respective centres, which sometimes are in use since many decades. For patient immobilization, many institutions build their own in-house developments for patient immobilisation [2, 4], which can lead to critical problems, if they are damaged or do not comply with medical device laws.

Nowadays, technological and technical improvements in modern radiotherapy, such as intensity modulated RT (IMRT) or volumetric modulated arc therapy (VMAT), have become widespread available in clinical practice. The theoretical advantages of IMRT/VMAT compared to 3-dimensional conformal RT (3DCRT) in TBI are: a better dose distribution within the target volume (in terms of homogeneity) and a dose reduction to healthy tissues and organs at risk, without the need for individualized external shielding [5]. Based on this background, we implemented a TBI technique using a standard linac-based set-up with VMAT. The main problem with using a linac-based set-up is that patients taller than 120 cm in body length cannot be treated in one position due to limited couch motion capacities of the linear accelerator [6]. Therefore, the patient is usually turned from a head-first position to a feet-first position to overcome this limitation [5]. In the present study we report on the development of a rotatable tabletop, which enables a safe and reproducible rotation of the patient during the VMAT TBI treatment, without an additional risk of overlapping fields.

## Material and methods

### Rotatable tabletop and patient set-up

**Fig. 1:**
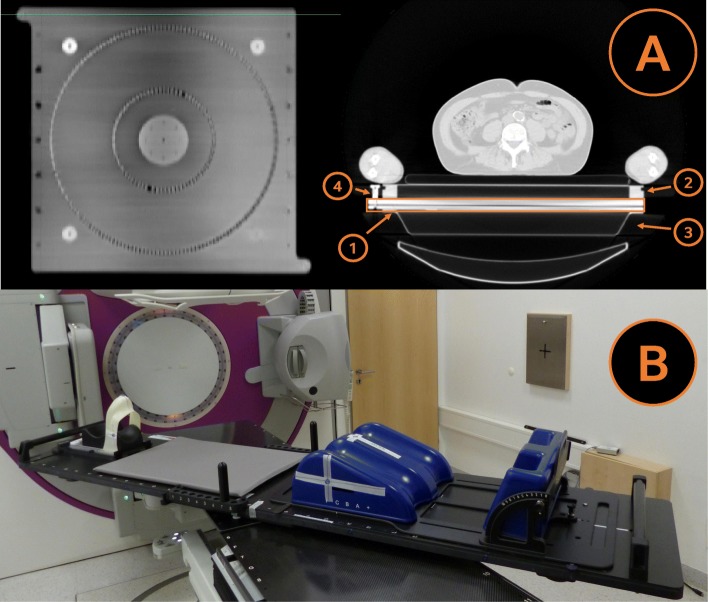
Coronal (**a** left) and axial (**a** right) CT-scan of the ball bearing rotational unit (1), holding the TBI-tabletop (2) on the linac couchtop (3). Carbon fixation buttons (4) lock the unit in head-first and feet-first positions to prevent unintended rotation. **b** shows the tabletop on the linac couchtop with integrated immobilization components

The newly developed rotatable tabletop has an integrated ball bearing that allows the rotation of the tabletop from a head-first position (HFS) to a feet-first position (FFS). The tabletop can be mounted on the CT or linac couchtop using an indexing system for better reproducibility. A CT-scan of the tabletop with the ball bearing is shown in Fig. 1. Furthermore, there are fixation buttons to hold the position of the rotation of the tabletop (see Figs. 1.4 and 2.1). The entire construction consists completely of carbon fiber. At the end of the tabletop, additional holding bars were attached for the staff, to enable an easy 180° rotation of the tabletop, within less than 10 s (see Fig. 2.2 and Additional file 2: Video S1).

**Fig. 2:**
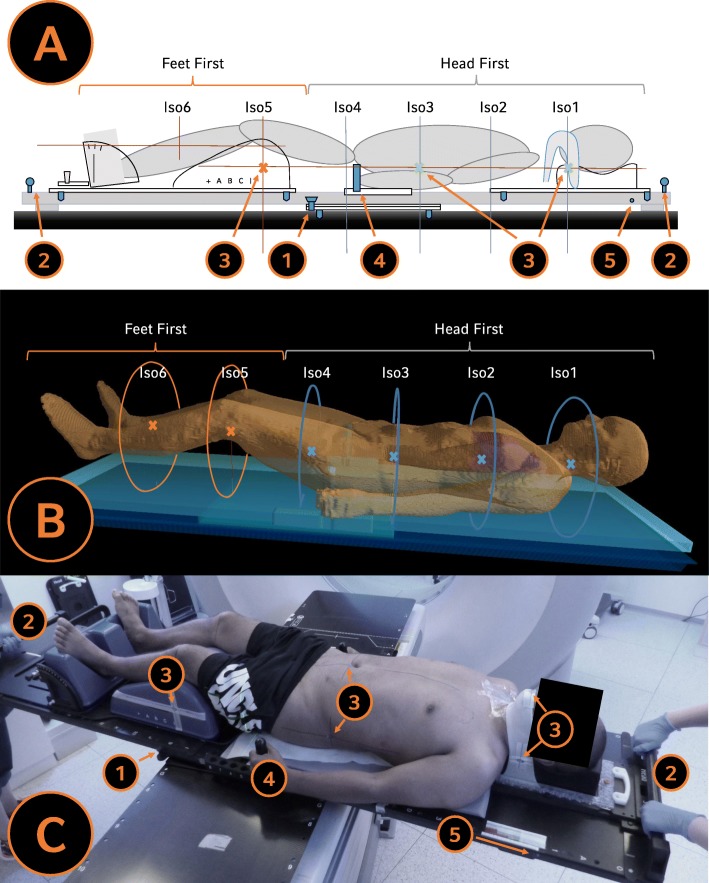
**a**-**c** Scheme and Example of patient setup on the rotatable tabletop. Image (**c**) shows the rotation from head-first to the feet-first-position. (1) Carbon fixation button. (2) Holding bars to facilitate rotation of the tabletop through staff (the patient weight is mainly on the rotational unit). (3) Markers for isocenters 1, 3 and 5. (4) Indexed arm bar. (5) Fixed marker for the baseline table coordinates of the “0” position (0-coordinate couch marker) of the iBEAM evo couchtable (Elekta AB, Stockholm, Sweden) at the linac, to enable the prediction of the final absolute table coordinates for a collision-free treatment delivery

In order to ensure a precise dose application, it is essential to immobilize the patient as comfortably and stable as possible. This allows to minimize the risk of unexpected movements during the treatment. For this purpose, various existing immobilization components (IT-V, Innsbruck, Austria) were integrated to ensure a consistent and reproducible patient immobilization for all different body parts. An example of the patient set-up is shown in Fig. 2. For head and thorax immobilization, the HeadSTEP system with the appropriate pillow in combination with an iCAST double layered chin mask was used. The open chin mask was used to mark the first isocenter (see Fig. 2.3) in order to avoid skin marks on the patient within the head and neck area. Moreover, the ProSTEP system was used for positioning of the lower abdomen and the legs. The system offers 17 longitudinal and 10 angular positions with an integrated indexing system to select the appropriate position for maximal patient comfort. For immobilization of the arms, an additional indexed arm bar was developed (see Fig. 2.4). A detailed description of all positions of the immobilization components was created during CT simulation to enable a reproducible patient positioning at the linac. Theoretically, the rotatable tabletop can also be used with other immobilization devices or vacuum cushions. The tabeltop is now commercially available through the manufacturer IT-V (Innsbruck, Austria).

### CT simulation

After immobilisation of the patient, the first step during CT simulation was to pre-define the isocenters for VMAT planning. For all patients, the lateral and vertical position of the 6–7 isocenters remained the same, whereas the longitudinal distance between the isocenters were chosen using a predefined scheme, depending on the patient size. Usually, 6 isocenters were used for patients up to a height of 180 cm: 4 in the head-first-supine (HFS) position and 2 isocenters in the feet-first-supine (FFS) position. In taller patents, we optionally added a 3rd isocenter in the FFS position. Three of these isocenters (“isocenter 1” on the chin mask, “isocenter 3” on the abdomen and “isocenter 5” on the ProSTEP) were marked during CT simulation (see Fig. 2.3). These isocenters were used to verify the patient position before the first treatment fraction. Additional CT markers were attached to the tabletop and represent the baseline table coordinates of the “0” position (0-coordinate couch marker) of the treatment couch (iBEAM evo Couchtop, Elekta AB, Stockholm, Sweden) at the linac; this marker was used to predict the final absolute table coordinates for a collision-free treatment delivery (see Fig. 2.5). CT simulation was first performed in a head-first position and afterwards the tabletop was rotated 180° to a feet-first position for a second CT scan, both with a slice thickness of 5 mm. A broad overlap region of both CTs was included, where visibility of the CT-markers of the isocenters 3 and 5 was required in both scans, to facilitate the image fusion in the planning software.

### Treatment planning

VMAT plans were optimized using the treatment planning system Monaco (version 5.11, Elekta AB, Stockholm, Sweden), which relies on the XVMC algorithm (X-ray voxel Monte Carlo [7]) for dose calculation using a grid spacing of 5 mm and a statistical uncertainty of 1% per calculation. All VMAT plans were generated for a 6MV Elekta Axesse linear accelerator (LINAC), equipped with an Agility multileaf-collimator (Elekta AB, Stockholm, Sweden).

**Fig. 3:**
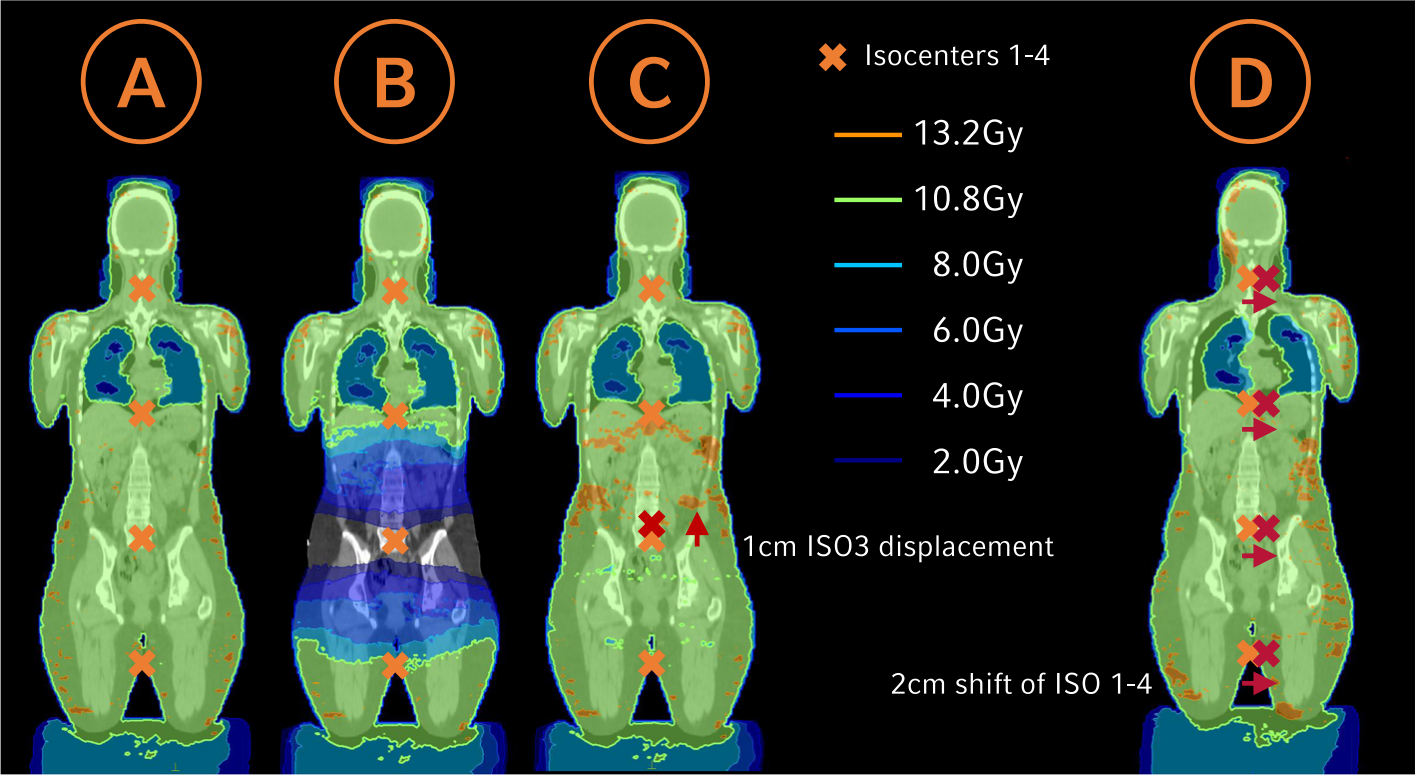
**a** Optimized VMAT-TBI plan. **b** Dose distribution without beam 3, illustrating the smooth and broad dose transition between the beams. **c** Robustness for small longitudinal isocenter displacements tested with a 1 cm cranial shift of isocenter 3. **d** Robustness check with all beams shifted 2 cm laterally

**Fig. 4:**
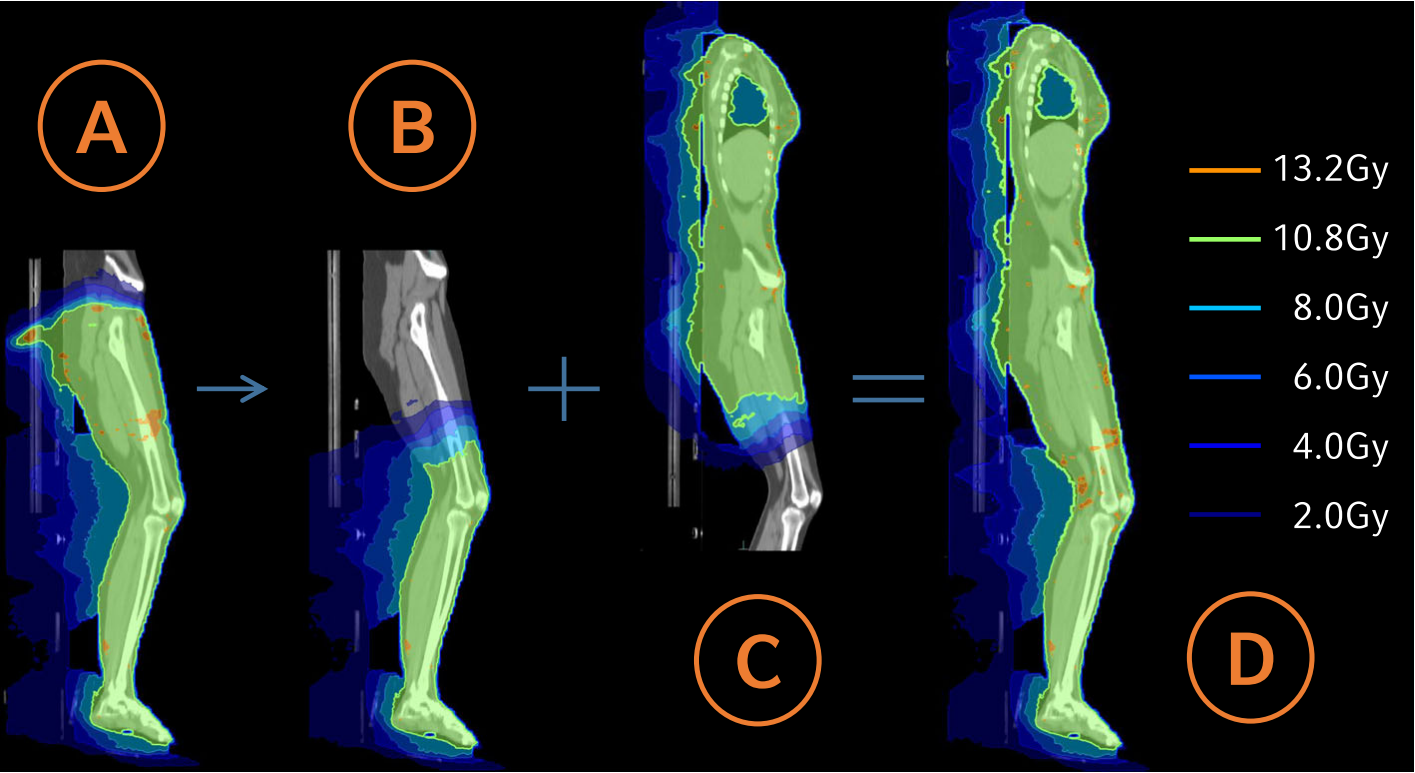
Planning principle of VMAT TBI. Optimization of FFS plan with an additional beam (**a**) that is deleted after optimization (**b**); this approach leads to a broad and smooth dose wash-out at the FFS-HFS junction area. The FFS dose is used as a bias for the optimization of the HFS plan (**c**), resulting in a homogenous summation dose in the whole body (**d**)

**Fig. 5:**
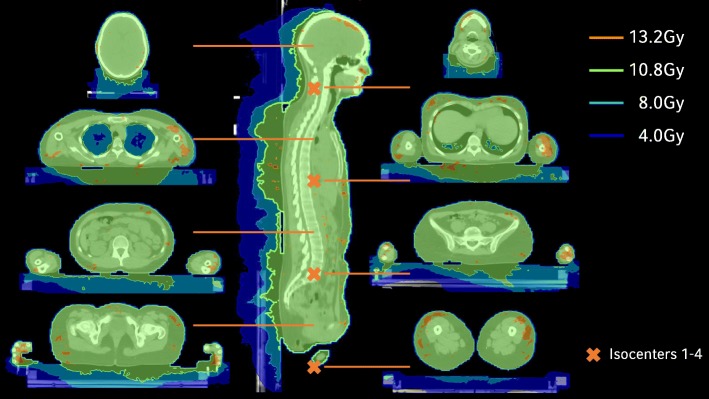
Examplary dose distribution of a VMAT TBI patient with axial slices in (right) and between (left) isocenter positions. Isodoses +/− 10% of the prescribed dose of 12Gy are shown

The treatment planning process included the following steps:

1. Marking of the 0-coordinate couch marker and the isocenters: CT markers were set as points of interest in Monaco, along with the other isocenters, which were not labeled with radiopaque markers during CT simulation (“isocenter 2”, “isocenter 4”, “isocenter 6”). The 0-coordinate couch marker was set as a treatment reference point to predict the absolute table coordinates of the isocenters for treatment delivery; these coordinates were kept within strict limits to prevent couch-gantry collisions.
2. Image fusion of HFS and FFS CT-scans: Image fusion was done manually in Monaco on bony structures (pelvis, femur and knees) and by a fine-tuning with the aid of the CT markers.
3. Optimization of the FFS treatment plan: The tolerance range for the prescribed dose was between 90 and 110%. As reported by Symons et al. [8], the PTV was cropped to a distance of 5 mm from the skin surface to increase homogeneity during dose optimization. Regarding the FFS plan, we focused on robustness and a smooth transition area between FFS/HFS beams. Monaco 5.11 automatically produces a broad dose transition of beams with different isocenters, as long as the collimator angles deviate from 0° and the junction areas are broad enough. Therefore, longitudinal isocenter shifts were chosen considerably smaller than the maximum field sizes: 23-35 cm for the FFS plans and 23-26 cm for the more critical region of the HFS treatment. In this way, a wide transition area could be created, without the use of additional help contours (see Fig. 3a+b), which makes the plan robust for small longitudinal isocenter displacements (see Fig. 3c). An additional beam was added at the caudal HFS isocenter position (“isocenter 4”) of the later HFS-treatment-plan during the optimization process, to create a smooth dose wash out at the FFS-HFS junction area. This auxiliary beam was deleted later (Fig. 4a+b). Robustness was further enhanced by the use of a 2.5 cm auto-flash margin (forces Monaco to open jaws near the surface of the patient) and optimization parameters that support the creation of large segments (large minimal segment width of 2 cm, large beamlet width of 0.5 cm, high smoothening of fluence).
4. Optimization of the HFS treatment plan using the optimized FFS-plan as a bias dose (see Fig. 4c+d): While other TPS have difficulties in junctioning two VMAT arcs that have been planned on two CTs with different treatment orientations [8], Monaco 5.11 allows the introduction of FFS-doses to a HFS treatment plan. As described in [5], the optimized FFS-dose can be used as a “bias” that is also included in the optimization process. Optimization parameters were the same as in the FFS-plan with regard to robustness, additional constraints were introduced to avoid hotspots in the abdominal and thorax region. For patients receiving a 10Gy or 12Gy dose protocol, mean lung dose was reduced below 8Gy or 10Gy, respectively, using a serial constraint for the lung volume. An example of the resulting dose distribution is shown in Fig. 5.
5. Transfer to the record-and-verify (R + V) system, including the absolute table coordinates of the isocenters: After approval, the plan was transferred to the R + V system. Absolute table coordinates for each beam isocenter were calculated with respect to the 0-coordinate couch marker and recorded manually to the R + V-system.
6. Re-calculation in a second TPS-system for quality assurance: For dose verification purposes, all plans were re-calculated in Oncentra Masterplan 4.5.2 (Elekta, Stockholm, Sweden) as a second independent TPS using a collapsed cone algorithm.
7. Robustness check: Robustness was checked by shifting beam isocenters in different directions and recalculating the plan. This was performed for individual isocenters, as well as for the whole isocenter set in a dummy run, before the treatment of the first patient at our institution. Nevertheless, the patient immobilization setup described above should reduce errors in vertical and longitudinal directions to an acceptable level. Considering the length of the treatment and a possible rotational error, a lateral displacement is the most likely. Therefore, as a standard robustness check for all patients, the final treatment plan was calculated with all beams shifted 2 cm laterally and evaluated whether dose distribution was still acceptable (see Fig. 3d).

### Treatment verification

During the first treatment, the patient was positioned at isocenter 1 using the markers that were placed on the chin mask during CT simulation. The resulting absolute table coordinates were compared to the predicted values that were recorded in the R + V system during the planning process. Slight differences are expected due to the weight of the patient (vertical shift), mechanical variability of the tabletop and the used CT-slice thickness of 5 mm. However, displacements should always be kept below 1 cm. The other coordinates of the HFS-isocenters were then corrected using the relative difference of the predicted coordinates of isocenter 1 to the actual couch coordinates, in order to keep the relative isocenter shifts at their planned values. Isocenter 1 and isocenter 3 were verified via cone-beam-CT (CBCT).

The FFS-isocenters were checked in a similar way, using the CT-marker of isocenter 5 as a reference and a CBCT of isocenter 5 for positioning verification. Additionally, an optical surface scan (Catalyst™, C-RAD AB, Sweden) was used to help position the patient’s legs. All isocenters were marked on the patient using skin marks. After verification of all three isocenters, beams were delivered sequentially with planned couch shifts. After FFS treatment, the tabletop was rotated 180° around the rotational unit to treat the HFS isocenters.

During the remaining treatment fractions, the patient was aligned using the skin marks and a room-laser system with no further image guidance, if no difficulties have occurred during the first treatment fraction. A more extensive image guidance is advisable if treatment planning includes more complicated dose prescriptions or organs at risk sparing of kidneys or eye lenses. Nevertheless, absolute table coordinates were checked routinely.

### Patient selection and endpoints

Twenty patients were analysed in the present study to evaluate the early feasibility of the method. Overall, this is a proof of concept study, with no thorough statistical analysis.

## Results

An overview of the patient and treatment characteristics of the first 20 patients can be found in Table 1. Overall, we treated 20 patients (10 female, 10 male) with a median age of 47 years (range: 21–60 years), all undergoing bone marrow transplantation for acute myeloid leukemia. Most patients (13/20) received a TBI dose of 4 Gy in 2 fractions. The mean number of applied monitor units (MU) was 6476 MU (range: 5366–7744 MU). During the first treatment fraction, usually 3 CBCTs (isocenter 1, 3, 5) were acquired in 85% of patients (17/20). A fourth CBCT was necessary, when there were major deviations and the patient was repositioned. The mean of absolute isocenter deviations detected at isocenter 1 (chin mask) were x = 0.24 ± 0.21 cm, y = 0.35 ± 0.23 cm and z = 0.13 ± 0.12 cm, at isocenter 3 (thorax): x = 0.35 ± 0.29 cm, y = 0.31 ± 0.22 cm and z = 0.28 ± 0.19 cm and at isocenter 5 (abdomen): x = 0.30 ± 0.28 cm, y = 0.39 ± 0.28 cm and z = 0.22 ± 0.15 cm (see Additional file 1: Table S1).

**Table 1:** Patient and treatment characteristics

Patient	diagnosis	gender	age (years)	total dose	single dose	fractions	patient height	patient weight	number of isocenters	longitudinal isocenter shift HFS	longitudinal isocenter shift FFS	MU applied	treatment time (h:m) fraction 1	no of CBCT	treatment time (h:m) fraction 2	Time difference Fx1 to Fx2
1	AML	m	43	4Gy	2Gy	2	178 cm	85 kg	6	25 cm	35 cm	7168MU	0:59	3	1:00	+ 0:01
2	AML	m	60	4Gy	2Gy	2	180 cm	82 kg	6	26 cm	34 cm	7744MU	0:53	3	0:31	0:22
3	AML	m	59	4Gy	2Gy	2	179 cm	74 kg	6	26 cm	34 cm	6066MU	1:07	4	0:31	0:36
4	AML	m	52	4Gy	2Gy	2	178 cm	88 kg	6	26 cm	34 cm	6999MU	0:46	3	0:35	0:11
5	AML	w	46	4Gy	2Gy	2	169 cm	52 kg	6	25 cm	32 cm	6363MU	0:51	3	0:27	0:24
6	AML	w	40	4Gy	2Gy	2	164 cm	67 kg	6	25 cm	30 cm	6150MU	0:51	3	0:45	0:06
7	AML	w	47	4Gy	2Gy	2	170 cm	80 kg	6	26 cm	29 cm	6531MU	1:04	3	0:28	0:36
8	AML	w	51	4Gy	2Gy	2	167 cm	71 kg	6	25 cm	30 cm	5868MU	0:52	3	0:44	0:08
9	AML	m	37	4Gy	2Gy	2	164 cm	47 kg	6	25 cm	30 cm	5782MU	0:53	3	0:50	0:03
10	AML	w	50	4Gy	2Gy	2	168 cm	78 kg	6	25 cm	30 cm	6666MU	0:51	3	0:50	0:01
11	AML	m	49	8Gy	2Gy	4	176 cm	98 kg	6	26 cm	32 cm	7033MU	1:17	3	1:05	0:12
12	AML	m	22	12Gy	2Gy	6	179 cm	70 kg	6	26 cm	32 cm	6036MU	0:52	3	0:38	0:14
13	AML	m	23	8Gy	2Gy	4	171 cm	70 kg	6	26 cm	34 cm	5778MU	0:49	4	0:28	0:21
14	AML	w	21	4Gy	2Gy	2	156 cm	39 kg	6	23 cm	23 cm	5366MU	0:51	3	0:31	0:20
15	AML	w	51	4Gy	2Gy	2	182 cm	94 kg	6	26 cm	34 cm	6916MU	1:15	3	0:30	0:45
16	AML	w	50	8Gy	2Gy	4	169 cm	63 kg	6	25 cm	30 cm	6870MU	1:28	3	0:40	0:48
17	AML	m	43	10Gy	2Gy	5	186 cm	89 kg	7	26 cm	26 cm (3)	7044MU	0:59	3	0:33	0:26
18	AML	w	42	4Gy	2Gy	2	167 cm	62 kg	6	25 cm	30 cm	6382MU	0:51	4	0:31	0:20
19	AML	w	59	8Gy	2Gy	4	165 cm	62 kg	6	25 cm	30 cm	5952MU	0:46	3	0:28	0:18
20	AML	m	22	12Gy	2Gy	6	180 cm	75 kg	6	26 cm	34 cm	6803MU	0:55	3	0:40	0:15
mean		(m:w/ 10:10)	43	(4Gy:8Gy:10Gy:12Gy/ 13:4:1:2)			172 cm	71 kg	(6:7 isoceters/ 19:1)	25 cm	31 cm	6476MU	0:57		0:38	0:20

Overall treatment time (OTT) was measured from the beginning of the first cone beam CT (during fraction 1) or the first beam (further fractions), respectively, until the end of the last beam. The time for initial patient setup was not taken into account, as these time points could not be extracted from the R + V system. Nevertheless, an additional 5 min for initial patient setup should be taken into account to assess the entire on-table-time. Regarding the first treatment fraction, the mean OTT (±standard deviation) was 57 ± 11 min. The OTT exceeded 1 h only in 5 patients, of whom the 2 patients with the longest OTT had to change linac unit during the fraction due to unrelated MLC problems. Since no additional image guidance using CBCT was used in fraction 2 of the same day, the OTT was reduced to mean 38 ± 11 min, which corresponds to a reduction of mean 20 ± 13 min.

## Discussion

There are various reports on TBI or total marrow irradiation (TMI) using volumetric modulated arc techniques [4–6, 9–17]. The main advantage is the improved dose homogeneity and the ability to individually spare organs at risk (OARs) as compared to conventional forward planned large field techniques [5]. Furthermore, this linac-based approach theoretically allows the use of standard equipment in the standard linac room, which is also used for conventional RT. Nevertheless, if reading most reports in detail, most techniques still use special tables, or even translational tables, or other self-made immobilization devices.

Mancosu et al. [18] have built a home-made dedicated immobilization system, called “All Body frame” for VMAT-based TMI. The system was composed of two rectangular plexiglas® boards and a dedicated head and shoulder board, where the patient was immobilized using three different thermoplastic masks (legs, thorax and abdomen). The boards were connected to each other and fixed to the couch. Nevertheless, between the treatment of the upper and lower part of the body, the patient had to get up from the couch and the immobilization device turned to a feet first position [6]. This procedure can be very time-consuming and prolong the overall treatment time. Similarly, Bao et al. [16] used a home-made immobilization system with integrated vacuum cushions and three thermoplastic masks for patient positioning. The authors describe that this system was turned 180° by two therapists into a FFS position. Nevertheless, it remains unclear if there was a dedicated rotational unit integrated into the system. The present study reports on the feasibility and early results of using the newly developed rotatable tabletop. Through the rotational unit, the patient can be turned 180° from a HFS to a FFS position within a few seconds, without the need of repositioning the patient (see video in Additional file 2). Moreover, the rotatable tabletop is completely built from carbon fiber, which adds no artefacts.

A study group from Calgary, Canada [9] implemented an extended SSD VMAT technique for TBI. In this case the treatment cannot be performed on the linac couch, as the linac couch is not capable of lowering the patient to a SSD of 175 cm. Therefore, the patient was positioned perpendicular to the conventional couch at a 90° couch angle on a customized bed. The table consisted of a modified massage table with a 1 cm acrylic spoiler to increase surface dose. Compared to this self-made solution, the rotatable tabletop presented in the current study, can be placed directly on the linac couch. This extended SSD technique was also implemented by other institutions using inversely modulated static open-field beams, which were delivered in an arc configuration [19], or more elaborated, using multiple consecutive modulated 5° subarcs in order to produce a more homogenous dose distribution [11]. Similarly, Jahnke et al. [13] used a single modulated sweeping arc version of this extended SSD technique, where the patient was treated AP/PA and a sweeping arc covered the entire body. Translational couch techniques hold the gantry still at 0° and the patients are translated on a couch, which is moved underneath the beam. Lately, also these techniques have been adapted for inverse planning to overcome dose heterogeneity due to variations in patient thickness, using couch speed variation or MLC aperture modulation [20, 21]. Taken together, all of these techniques used customized couches, positioned on the room floor to reach the extended SSD.

Springer et al. [10] developed an inversely planned arc technique, where the patient was positioned on the linac couch, similar to the present study. Multiple beam isocentric planning was used (8 isocenters) and the inverse optimization provided smoothing at the junctions. The patient was positioned on a vacuum mattress placed into a custom made wooden box, in order to guarantee a stable mattress. For the head-, neck- and shoulder-regions thermoplastic masks were used. After treatment of the 4 proximal segments, the patient was rotated 180° to irradiate the FFS segments. Overall, the treatment duration was 1.5 h per fraction (of note: 2 h for the first fraction). This was significantly longer than the OTT reported in the present study and might be due to the need of repositioning the patient and more isocenters than in the present study.

Ouyang et al. [4] developed a similar rotational immobilization system as in the present study, called “IRIS” (indexed rotatable immobilization system). Nevertheless, the IRIS was an in-house developed patient immobilisation tool, made out of wood panels, and is not commercially available. The newly developed rotatable tabletop presented in this study, consists completely of carbon fiber, including the ball bearing within the base plate of the rotation unit and complies with medical device laws. Hopefully, this new development will make the rotational TBI techniques available to more institutions as it overcomes one of the most important limitations of VMAT-TBI: the limited cranial-caudal couch shift capacities of the linac.

Of note, also the use of a TomoTherapy® system (Accuray Inc., Sunnyvale, USA) encounters the need to reposition the patients due to the limited translation length of the couch, allowing a treatment lengths of approximately 145 cm [22, 23]. In case of patients exceeding 145 cm in body length, TBI using tomotherapy is usually delivered using two separate plans. After treatment of the upper body, the lower body part is usually delivered after repositioning in a feet-first-position using a second tomotherapy plan [22], or using ap/pa fields on a regular Linac [24].

## Conclusion

The main focus of the development of this rotatable tabletop was on practicability in clinical routine. After implementation of the VMAT-TBI technique, the tabletop has been used successfully in daily clinical practice and helped to keep the treatment times at an acceptable level. The easy and reproducible rotation of the patient on the treatment couch using the rotatable tabletop is time-efficient and overcomes the need of repositioning the patient after turning from a HFS to a FFS position. Furthermore, this procedure can also help to reduce positional errors to a minimal level. Isocenter selection can be considered a central point during the planning process and the integration of those isocenters to the R + V system via absolute predicted couch coordinates, proved to be crucial for the following reasons:

- It supports a fast patient setup
- Couch-Gantry collisions are avoided due to clear guidelines for allowed isocenter coordinates
- Incorrect isocenter shifts can be avoided
- Beam mix-up can be avoided, as beams are approved for fixed table positions

Other advantages of the tabletop are that due to the carbon fibre material of the whole construction, the tabletop can be easily integrated into dose calculations and no structures need to be avoided for the incoming beams. Moreover, during an unexpected linac failure, the patient can easily switch to another linac unit, as no special additional equipment is needed.

## Supplementary information

**Additional file 1:** **Table S1.** Exemplary translational Cone-Beam CT setup deviations (in centimeter) of the 3 different isocenters during the first treatment fraction along the x (lateral), y (anterior-posterior) and z (inferior-superior) axis.

**Additional file 2: Video S1.** Exemplary video showing the 180° rotation from a HFS to a FFS position within a few seconds, without the need of repositioning.

## Data Availability

Not applicable.
