# Supplementary material for: Novel rotatable tabletop for total-body irradiation using a linac-based VMAT technique
Source: Radiat Oncol. 2019 Dec 30;14:244. doi: 10.1186/s13014-019-1445-3 (PMC6937701; doi:10.1186/s13014-019-1445-3)
Supplement: Supplementary file 1 — Additional file 1: Table S1. Exemplary translational Cone-Beam CT setup deviations (in centimeter) of the 3 different isocenters during the first treatment fraction along the x (lateral), y (anterior-posterior) and z (inferior-superior) axis. [file 13014_2019_1445_MOESM1_ESM.docx]

**Additional file 1**

**Table S1:** Exemplary translational Cone-Beam CT setup variations (in centimeter) of the 3 different isocenters during the first treatment fraction in centimeters along the x (lateral), y (anterior-posterior) and z (inferior-superior) directions.

|  | **CBCT Head** | | | **CBCT Thorax** | | | **CBCT Abdomen** | | |
| --- | --- | --- | --- | --- | --- | --- | --- | --- | --- |
| **Patient** | **x** | **y** | **z** | **x2** | **y2** | **z2** | **x3** | **y3** | **z3** |
| 1 | -0.23 | -0.25 | 0,15 | -0.50 | 0.67 | 0.14 | 0.66 | -0.73 | 0.20 |
| 2 | 0.04 | -0.78 | 0.20 | 0.05 | -0.31 | -0.35 | -0.12 | 0.43 | -0.18 |
| 3 | -0.36 | -0.74 | -0.16 | -0.20 | -0.05 | -0.71 | -0.29 | 0.05 | 0.22 |
| 4 | 0.19 | -0.17 | 0.07 | -0.13 | 0.72 | -0.22 | 0.26 | -0.51 | 0.38 |
| 5 | 0.12 | -0.18 | 0.06 | -0.07 | 0.21 | -0.22 | -0.19 | -0.41 | 0.45 |
| 6 | -0.55 | 0.34 | 0.17 | -0.08 | 0.40 | -0.34 | 0.00 | -0.74 | 0.29 |
| 7 | -0.07 | -0.04 | 0.03 | -0.48 | 0.27 | -0.07 | -0.79 | -0.01 | 0.16 |
| 8 | -0.10 | 0.07 | -0.25 | 1.11 | 0.34 | -0.03 | -0.27 | -0.78 | 0.15 |
| 9 | -0.56 | -0.19 | 0.16 | -0.07 | 0.41 | -0.12 | -0.04 | -0.71 | 0.10 |
| 10 | -0.21 | -0.64 | 0.00 | -0.52 | 0.03 | -0.46 | 0.80 | -0.51 | 0.59 |
| 11 | -0.01 | -0.27 | -0.01 | 0.56 | 0.09 | 0.11 | 0.40 | -0.08 | 0.20 |
| 12 | -0.05 | -0.30 | 0.03 | 0.09 | 0.23 | -0.27 | 0.13 | 0.10 | 0.09 |
| 13 | 0.26 | -0.60 | 0.46 | 0.32 | -0.09 | -0.23 | 0.54 | 0.37 | 0.17 |
| 14 | 0.22 | 0.36 | 0.22 | -0.47 | 0.59 | -0.43 | 0.08 | -0.43 | 0.07 |
| 15 | 0.68 | -0.32 | 0.04 | -0.55 | 0.24 | -0.51 | 0.00 | 0.03 | -0.06 |
| ***mean*** | ***-0.04*** | ***-0.25*** | ***0.07*** | ***-0.06*** | ***0.25*** | ***-0.25*** | ***0.08*** | ***-0.26*** | ***0.19*** |
| ***mean of absolute values*** | ***0.24*** | ***0.35*** | ***0.13*** | ***0.35*** | ***0.31*** | ***0.28*** | ***0.30*** | ***0.39*** | ***0.22*** |
| ***standard deviation of absolute values*** | ***±0.21*** | ***±0.23*** | ***±0.12*** | ***±0.29*** | ***±0.22*** | ***±0.19*** | ***±0.28*** | ***±0.28*** | ***±0.15*** |
| ***max of absolute values*** | ***0.68*** | ***0.78*** | ***0.46*** | ***1.11*** | ***0.72*** | ***0.71*** | ***0.80*** | ***0.78*** | ***0.59*** |
